# H3K18 Lactylation in Degenerated Nucleus Pulposus Cells Suppresses CD248 Through Transcriptional and Post‐Translational Modifications to Promote Apoptosis and Extracellular Matrix Degradation

**DOI:** 10.1111/acel.70810

**Published:** 2026-11-10

**Authors:** Yiming Ma, Tuo Shao, Qichang Gao, Jiaao Gu, Wenbo Zhan, Shuning Han, Song Li, Zhange Yu

**Affiliations:** ^1^ Department of Spinal Surgery The First Affiliated Hospital of Harbin Medical University Harbin Heilongjiang Province China; ^2^ Department of Central Laboratory The First Affiliated Hospital of Harbin Medical University Harbin Heilongjiang Province China; ^3^ Hebei Medical University Third Hospital Shijiazhuang City Hebei Province China; ^4^ Mudanjiang Medical University Mudanjiang Heilongjiang Province China

**Keywords:** apoptosis, intervertebral disc degeneration, lactylation, nucleus pulposus degeneration

## Abstract

Intervertebral disc degeneration (IVDD) is mainly driven by nucleus pulposus (NP) degeneration, a process associated with increased lactate accumulation. This study explored the role of histone H3K18 lactylation (H3K18la) in NP degeneration. Degenerated NP cells showed increased levels of lactate and lactylation compared to normal cells. Adding external lactate and glycolytic inhibitor (2‐DG) positively and negatively regulated lactylation levels, respectively. Subsequently, we discovered that lactate regulates CD248 at both transcriptional and protein levels through H3K18la. IP/MS, ChIP‐WB, and ChIP‐reChIP‐qPCR showed that H3K18la recruits SMARCA5 to co‐enrich at the CD248 promoter, which suppresses CD248 mRNA expression. At the protein level, lactate promotes CD248 degradation through the ubiquitin–proteasome pathway (UPP). By knocking down and overexpressing CD248, it was found that it could improve the apoptosis of NP cells and the degradation of extracellular matrix (ECM) by activating the Hedgehog pathway. Our study shows that lactate regulates CD248 at both the transcriptional and protein levels through H3K18la, resulting in dual‐level suppression of CD248. These findings define a lactate–H3K18la–CD248 regulatory axis in IVDD.

## Graphical abstract

The high lactate levels in degenerated intervertebral discs promote lactylation of NP cells. H3K18la inhibits the synthesis of CD248 mRNA by recruiting SMARCA5 and promotes the protein degradation of CD248 through the ubiquitin–proteasome pathway, thereby inducing apoptosis of NP cells and degradation of ECM.

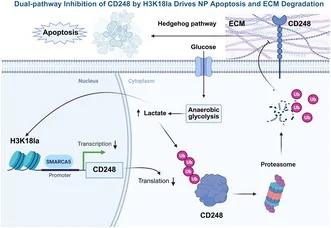

## 1 Introduction

Intervertebral disc degeneration (IVDD) is one of the core pathological processes of spinal degenerative diseases (Diwan and Melrose 2023). The intervertebral disc primarily consists of the annulus fibrosus, cartilage endplate, and nucleus pulposus (NP) (Bibby et al. 2001). NP serves as the primary shock‐absorbing component of the disc (Roberts et al. 2006; J. P. Urban and McMullin 1988). The pathogenesis of IVDD is complex, involving multifactorial interactions such as extracellular matrix (ECM) degradation, NP cell apoptosis, activation of inflammatory responses, and abnormal mechanical loading (Bai et al. 2020; He et al. 2021; Zhou et al. 2022). Current research points out that NP cell death (de Schepper et al. 2010), ECM metabolic dysregulation (Jo and Chae 2021), and secondary inflammation (Ding et al. 2013) are the three primary contributors to IVDD.

It has been reported that lactate levels are higher in degenerated human NP tissues (Zhao et al. 2021). As the largest avascular organ in the human body, intervertebral discs exist in a long‐term hypoxic environment, their main energy supply comes from anaerobic glycolysis. Over time, this metabolic process leads to gradual accumulation of lactate as the end product of glycolysis (J. P. G. Urban et al. 2004). NP cells are very sensitive to extracellular pH, and acidic environments can inhibit the production rate of ECM, thus accelerating disc degeneration (Ishihara and Urban 1999). For a long time, lactate was only seen as a metabolic waste. The ways in which lactate changes affect disease development are still unclear. In recent years, metabolite‐induced histone post‐translational modifications (PTMs) have become more and more important in epigenetic research. These modifications function by changing nucleosome interactions or recruiting non‐histone proteins, including acetylation, glycosylation, ubiquitination, methylation, succinylation, and phosphorylation. Most of these modification substrates come from metabolic processes and may provide new strategies for various diseases.

D. Zhang et al. (2019) first demonstrated that lactate can covalently bind to lysine residues on histones, forming lactate‐lysine (Kla) structures. This identified lactylation as a new epigenetic PTM. Subsequent studies have revealed significant roles of lactylation across multiple research fields (Wang, Yang, et al. 2022; Wang, Wang, et al. 2022; Yu et al. 2021). However, the role of lactylation in IVDD and NP degeneration is still not clear. Therefore, we hypothesize that the increased accumulation of lactate in degenerated NP cells induces histone lactylation, which is the basis of our current research.

In this study, we found histone lactylation in degenerated NP cells and verified its modification sites. Overall, histone lactylation inhibited NP cell proliferation and migration while promoting apoptosis and ECM degradation. Glycolytic inhibitor 2‐DG suppressed lactylation and improved these pathological processes. After H3K18 point mutation, we observed reduced global lactylation levels in NP cells. After confirming CD248 as a downstream target of H3K18la, we established that CD248 regulates NP cell apoptosis and ECM synthesis through the Hedgehog pathway. Our findings first revealed that H3K18la recruits SMARCA5 as a transcriptional repressor to inhibit CD248 expression. Moreover, lactate regulates CD248 through multiple mechanisms, including ubiquitin–proteasome‐mediated protein degradation. These results provide new experimental evidence for developing treatment strategies for NP degeneration.

## 2 Materials and Methods

### 2.1 Cell Culture

NP tissues were collected from patients undergoing spinal surgery at the First Affiliated Hospital of Harbin Medical University between 2022 and 2024. Tissues were classified according to the Pfirrmann grading system (Grades II–V). Grade II degenerated NP tissues were obtained from patients with traumatic lumbar fractures requiring internal fixation or scoliosis. Grades III–V degenerated NP tissues were collected from patients with lumbar degenerative diseases. All specimens were rinsed repeatedly with D‐Hanks solution until blood‐free. Peripheral bone and annulus fibrosus were carefully removed using ophthalmic scissors, and NP tissues were cut into small pieces. Tissue digestion was done first with 0.25% trypsin and then with Type II collagenase. The cells were resuspended in DMEM/F12 medium (KGL1202‐500, KeyGEN BioTECH, Nanjing, China) supplemented with 10% fetal bovine serum (3021A, Umedium, Hefei, China) and seeded evenly into T25 flasks. Cells were cultured at 37°C with 5% CO_2_ and saturated humidity, with medium changes every 3 days.

The immortalized human NP cells line was purchased from Procell (CP‐H097, Wuhan, China) in December 2022 and cultured using the same method as the primary human NP cells. All cells (including both human primary NP cells and the established cell line) were maintained in media containing mycoplasma removal agents (BL591B, biosharp, Hefei, China) to prevent contamination. The absence of mycoplasma in all cultures used for experiments was verified prior to use.

All patients had complete clinical and radiological documentation. This study was approved by the Ethics Committee of the First Affiliated Hospital of Harbin Medical University (No. 202573) and conducted in accordance with the Declaration of Helsinki. Written informed consent was obtained from all participants.

### 2.2 Untargeted Metabolomics

Human primary cells were quickly frozen in liquid nitrogen and sent to Metware (Wuhan, China) for untargeted metabolomics analysis. All samples were analyzed using two LC/MS methods. One part was analyzed under positive ion conditions, and the other part under negative ion conditions. The elution gradient was the same as that of the positive mode. All the methods alternated between full scan MS and data‐dependent MSn scans using dynamic exclusion.

### 2.3 Measurement of Lactate Levels

After centrifugation at 12,000 *g* for 10 min, the supernatant from homogenized NP tissue was collected. The lactate content in NP tissue was measured according to the instructions of the assay kit (A019‐2‐1, Nanjing Jiancheng Bioengineering Institute, Nanjing, China). Tissue samples were weighed and homogenized in ice‐cold physiological saline at a weight (g)‐to‐volume (mL) ratio of 1:9 using mechanical homogenization. The homogenate was centrifuged at 4000 rpm for 10 min, and the resulting supernatant was collected for analysis. Absorbance at 530 nm was measured using a microplate reader (SpectraMax iD5, Molecular Devices, USA).

### 2.4 Protein Extraction and Western Blot

Cells or tissues were lysed using RIPA buffer (Beyotime Biotechnology, Shanghai, China) containing protease and phosphatase inhibitors (Beyotime). Total protein content was quantified using the BCA assay (Servicebio, Wuhan, China). Denatured proteins were separated by SDS‐PAGE and transferred onto polyvinylidene fluoride (PVDF) membranes (Millipore, Saint Charles, MO, USA). After blocking with rapid protein‐free blocking buffer (G2052, Servicebio) for 10 min at room temperature, PVDF membranes were incubated with primary antibodies overnight at 4°C. Subsequently, membranes were incubated with corresponding secondary antibodies for 1 h at room temperature and washed three times with TBST. Protein bands were visualized using an enhanced chemiluminescence (ECL) kit (Biosharp) under the ChampChemi chemiluminescence system (Sinsage, Beijing, China). Band intensities were analyzed using ImageJ software, with relative protein levels normalized to β‐actin. The antibodies used in the experiment are shown in the additional Supplementary Antibodies.

### 2.5 RNA Interference and Overexpression

siRNA was dissolved to a concentration of 20 μM, and RNA interference was performed using siRNA‐mate plus according to the manufacturer's instructions (GenePharma, Shanghai, China). RNA was collected 24 h post‐transfection, and protein was collected after 48 h. The siRNA sequences were as follows: Si‐CD248‐1: CCCCUAUGAUCUCAACCAATT; UUGGUUGAGAUCAUAGGGGTT, Si‐CD248‐2: CCACUCAUUUGCCUGGAAUTT; AUUCCAGGCAAAUGAGUGGTT. Si‐SMARCA5‐1: GGACCGAGCACAUAGAAUUTT; AAUUCUAUGUGCUCGGUCCTT, and Si‐SMARCA5‐2: CCACCUUAUACAACAGAUATT; UAUCUGUUGUAUAAGGUGGTT.

The H3K18 WT and H3K18R plasmids were constructed by OBIO (Shanghai, China), while the CD248‐OE plasmid was constructed by GenePharma. Transfection was performed using Lipo8000 transfection reagent (C0533, Beyotime) according to the manufacturer's instructions. RNA was collected 24 h post‐transfection, and protein was harvested after 48 h.

### 2.6 mRNA‐Seq

Raw sequencing data was first filtered by fastp (v0.23.2). Clean Reads were further treated with in‐house scripts to eliminate duplication bias introduced in library preparation and sequencing. Reads in the same cluster were compared to each other by pairwise alignment, and then reads with sequence identity over 95% were extracted to a new sub‐cluster. After all sub‐clusters were generated, multiple sequence alignment was used to get one consensus sequence for each sub‐clusters. After these steps, any errors and biases introduced by PCR amplification or sequencing were eliminated. The sequences were used for standard mRNA‐seq analysis. Differentially expressed genes between groups were identified using the edgeR package (version 3.40.2), and a *p*‐value cutoff of 0.05 and a fold‐change cutoff of 2 were used. Gene Ontology (GO) analysis and Kyoto Encyclopedia of Genes and Genomes (KEGG) enrichment analysis for differentially expressed genes were both implemented by KOBAS software (version: 2.1.1) with a *p*‐value cutoff of 0.05 to determine a statistically significant enrichment.

### 2.7 Cell Counting Kit‐8 Assay

Cell Counting Kit‐8 (CCK‐8; P‐CA‐001, Procell) was used to measure cell proliferation. Cells were seeded in 96‐well plates at a density of 3 × 10^3^ cells per well. After treatment, 10 μL of CCK‐8 solution was added to each well, followed by incubation for 2 h. Absorbance at 450 nm was measured using a microplate reader (SpectraMax iD5). Finally, the number of viable cells was plotted to reflect the proliferation rate.

### 2.8 5‐Ethynyl‐2′‐Deoxyuridine (EdU) Assay

The EdU assay was performed using the EdU Detection Kit (C0071S, Beyotime). Briefly, cells were incubated with freshly prepared EdU working solution (10 μM) for 24 h. After EdU labeling, cells were fixed with fixative solution at room temperature for 15 min. After washing three times with washing buffer, cells were permeabilized with permeabilization buffer at room temperature for 15 min and washed again. The Click Additive Solution was then added and incubated with cells for 30 min at room temperature in the dark. Finally, cells were mounted with antifade mounting medium containing DAPI and imaged under a fluorescence microscope (ECLIPSE Ti2, Nikon, Japan).

### 2.9 Wound Healing Assay

NP cells were evenly seeded in 6‐well plates. A straight wound was created perpendicular to the reference line using a 200 μL pipette tip, followed by PBS washes to remove dislodged cells. The cells were then cultured in medium containing 5% serum at 37°C with 5% CO_2_ for 24 h. Wound healing was imaged using a microscope (ECLIPSE Ti2, Nikon).

### 2.10 Flow Cytometry

Cells were harvested using EDTA‐free trypsin (C0205, Beyotime), centrifuged, and washed twice with PBS. After discarding the supernatant, cells were resuspended in 100 μL of 1× Annexin V Binding Buffer, followed by the addition of 2.5 μL Annexin V‐FITC Reagent and 2.5 μL PI Reagent. The mixture was incubated at room temperature in the dark for 15–20 min. Subsequently, 400 μL of diluted 1× Annexin V Binding Buffer was added, and the samples were mixed thoroughly. Unstained cells served as the negative control, while FITC and PI only stained cells were used to set the flow cytometer voltage and compensation. All staining reagents were from the Annexin V‐FITC/PI Apoptosis Kit (E‐CK‐A211, Elabscience, Wuhan, China). Apoptosis was analyzed using a flow cytometer (Apogee Universal, England) with 488 nm excitation, detecting fluorescence in the FITC and PI channels. Data were analyzed using FlowJo software (Version 10.8.1).

### 2.11 Immunofluorescence

Glass slides coated with poly‐l‐lysine were placed in 24‐well plates, and NP cells were seeded into the plates, followed by incubation at 37°C. After 24 h, cells were fixed with 4% paraformaldehyde and permeabilized with 0.1% Triton X‐100. Following PBS washes, cells were blocked with 10% goat serum (G1208, Servicebio) for 1 h. Cells were then incubated with primary antibodies overnight at 4°C, followed by incubation with fluorescent secondary antibodies (A0453, Beyotime) for 2 h at room temperature. Finally, cells were mounted with antifade mounting medium containing DAPI (P0131, Beyotime) and imaged using a fluorescence microscope (ECLIPSE Ti2, Nikon).

### 2.12 Reverse Transcription Quantitative Polymerase Chain Reaction (RT‐qPCR)

Total RNA was extracted from cell samples using a column‐based RNA extraction kit (BSC52M1, Bioer Technology, Wuhan, China) and quantified with a NanoDrop spectrophotometer (Thermo Fisher). RNA was reverse‐transcribed into cDNA. All primers were synthesized by Sangon Biotech (Shanghai, China). Each cDNA sample was prepared in a 20 μL reaction system. Data were analyzed using the 2^−ΔΔCT^ method. The primer sequences used were as follows: Human CD248 (F: 5′‐GGACAGAGAGCCACAGATACCC‐3′, R: 5′‐GTGACGGAGAGCACTGAGGAG‐3′), human β‐actin (F: 5′‐ATTGCCGACAGGATGCAGAA‐3′, R: 5′‐CGGACTCGTCATACTCCTGC‐3′), and human SMARCA5 (F: 5′‐ACAAACAACTGCCTTGGGGA‐3′, R: 5′‐TTTGCATTTTGCTGAGGCCC‐3′).

### 2.13 Chromatin Immunoprecipitation (ChIP)‐qPCR

The procedure was performed according to the ChIP kit instructions (P2078, Beyotime). NP cells were cultured in 10 cm dishes and fixed with 37% formaldehyde at 37°C for 10 min. Cells were washed with PBS containing PMSF and centrifuged at 1200 *g* for 2 min at 4°C. Genomic DNA was sheared by sonication (10% power, 10 s on/10 s off, 3 cycles). The sonicated samples were centrifuged at 12,000 *g* for 5 min at 4°C, and the supernatant was collected.

The supernatant was divided into Input, IgG, and IP groups. The IgG group was incubated with IgG antibody (A7016, Beyotime), while the IP group was incubated with the primary antibody. Then, they were gently rotated at 4°C overnight. The next day, Protein A/G was added to precipitate the target protein or complexes, followed by continued rotation at 4°C. Samples were then washed according to the manufacturer's protocol, and 5 M NaCl was added to the washed samples. The protein–DNA crosslinks were reversed by heating at 65°C for 4 h. The resulting DNA was purified using a DNA purification kit (D0033, Beyotime) and subjected to qPCR.

For ChIP‐reChIP, the first‐round ChIP eluate was incubated overnight with a reChIP antibody, followed by the same steps as the first‐round ChIP.

ChIP primers targeting the CD248 promoter region were as follows: Primer 1 (F: 5′‐AGGGCAGTTTACTGAAGGGC‐3′, R: 5′‐GCGGATTAACCCATGCACAC‐3′), Primer 2 (F: 5′‐TCTGAGTCTCTGGGAAGCGA‐3′, R: 5′‐GAGTCAGGGGGTGGGAAAAG‐3′), Primer 3 (F: 5′‐TTGTAGCGAGGGACATGAGC‐3′, R: 5′‐GCAACTCATCTCCGAGGTCC‐3′), and Primer 4 (F: 5′‐CCAGGGTCCTCTCTCAACCA‐3′, R: 5′‐TTGGGTAAGGTTGACACCCC‐3′).

### 2.14 Chromatin Immunoprecipitation‐Western Blot

The preceding steps were performed as described in Method 13. After eluting the ChIP products, 20 μL of 1× loading buffer (P0015A, Beyotime) was added, followed by boiling the samples at 100°C. The proteins were separated by SDS‐PAGE and transferred onto PVDF membranes. Subsequent steps were identical to Section 2.4. The antibodies used for ChIP‐WB were as follows: anti‐H3K18la (PTM‐1427RM, PTM Bio, Hangzhou, China), anti‐SMARCA5 (13066‐1‐AP, Proteintech, Wuhan, China), anti‐P53 (SA39‐07, HUABIO, Hangzhou, China), and anti‐Histone H3 (17168‐1‐AP, Proteintech).

### 2.15 Co‐Immunoprecipitation (Co‐IP) Assay

Briefly, cells were collected and lysed with RIPA buffer (Beyotime), followed by centrifugation at 12,000 rpm for 15 min at 4°C. The supernatant was divided into Input, IgG, and IP groups. The IP group was incubated with primary antibodies overnight at 4°C. Protein A/G Magnetic Beads (HY‐K0202, MCE, USA) were then added and gently mixed for 2 h at 4°C. The precipitates were washed five times with RIPA buffer, loaded, and analyzed by Western blotting. The total harvested proteins were loaded and separated on a 7.5% sodium dodecyl sulfate–polyacrylamide gradient gel. Subsequent steps were identical to those described in Section 2.4. The primary antibodies used in this study were as follows: anti‐H3K18la (PTM‐1427RM, PTM Bio) and anti‐CD248 (18160‐1‐AP, Proteintech).

### 2.16 Co‐Immunoprecipitation Assay and Mass Spectrometry (MS)

Mass spectrometry analysis was performed by SpecAlly Life Technology Co. Ltd. (Wuhan, China). Magnetic beads from Method 15 were collected, and the samples were incubated with reaction buffer at 95°C for 10 min to complete protein denaturation, reduction, and alkylation. After centrifugation, the supernatant was collected and diluted with an equal volume of ddH_2_O. Trypsin was added at a 1:50 (enzyme‐to‐protein) mass ratio, followed by overnight digestion with shaking at 37°C. The next day, trifluoroacetic acid (TFA) was added to terminate the digestion, and samples were centrifuged at 12,000 *g*. The supernatant was desalted using homemade SDB columns, vacuum‐dried, and stored at −20°C.

LC–MS/MS analysis was performed using a Thermo Ultimate 3000 RSLC nano system. Raw data were processed with MaxQuant software (v2.2.0.0) using the built‐in Andromeda search algorithm. Bioinformatics analysis and visualization were conducted using R. Protein quantification values were log2‐transformed, and missing values were imputed from a normal distribution representing the detection limit. Proteins with valid quantification values in at least 50% of samples within any group were included for comparative analysis. Differentially expressed proteins (DEPs) were identified based on *t*‐test *p*‐values and fold change (FC) thresholds.

### 2.17 Rat Model of Intervertebral Disc Degeneration

Sprague Dawley (SD) rats were purchased from the Experimental Animal Research Center of the First Affiliated Hospital of Harbin Medical University. All experimental procedures were approved by the Institutional Animal Care and Use Committee of the First Affiliated Hospital of Harbin Medical University (No. 202224). SD rats were randomly divided into four groups: Control group (no intervention), IVDD group (puncture‐only treatment), IVDD+AAV9‐OE‐NC (puncture followed by intradiscal delivery of 2 μL AAV9‐OE‐NC), and IVDD+AAV9‐OE‐CD248 (puncture followed by intradiscal delivery of 2 μL CD248‐overexpressing AAV9); the AAV9 adenovirus‐associated virus overexpressing CD248 was ordered from WZ Biosciences Inc. (Jinan, China). Male SD rats aged 6–8 weeks were anesthetized via intraperitoneal injection of 0.5% sodium pentobarbital. Under anesthesia, intervertebral discs (Co7/8, Co8/9, and Co9/10 in each animal's tail) were punctured using a sterile 23G needle. Under ultrasound guidance, the location of the intervertebral disc was determined, and then the needle was inserted through the epidermis and annulus fibrosus of the rat's tail vertebra into the central area of the NP. Then, the needle was stopped from further insertion, rotated 360 degrees, and held for 30 s to induce degeneration. In the IVDD+AAV9‐OE‐NC and IVDD+AAV9‐OE‐CD248 groups, a 10 μL injection syringe was fixed to a micro‐injection pump (M5258, Changsha Maiyue Biotechnology Co. Ltd., 2 μL/min), and AAV9 (2 μL per intervertebral disc) was injected immediately after the puncture. After the injection is completed, stay for 1 min to ensure that the liquid does not leak out. After the surgery, the animals recovered in a warm and ventilated cage.

### 2.18 Histopathological Staining

Rat tail intervertebral discs were harvested 6 weeks post‐treatment. After washing with PBS, samples were fixed in 4% paraformaldehyde (Biosharp, 143174) for 48 h, decalcified in 5% formic acid (G1107, Servicebio) for 1 week. After that, the samples were dehydrated, embedded in paraffin, and cut into 5 μm‐thick slices using a microtome (HM355S, Thermos Fisher, USA). H&E staining was used to evaluate the overall structure of the discs and the number of cells in each part, including the annulus fibrosus, NP, and cartilage endplate. Safranin O (S‐O) staining was used to assess proteoglycan and collagen distribution. Masson trichrome staining was used to tell collagen fibers apart from other tissue components, which helped evaluate NP fibrosis. These staining methods enabled comprehensive assessment of IVD degeneration under different interventions. Stained sections were imaged under a microscope (ECLIPSE Ti2, Nikon). All staining reagents were purchased from Servicebio. For the histopathological staining, we employed a new rat IVDD scoring system (Lai et al. 2021). This system comprehensively assesses the degree of degeneration of the intervertebral discs in the tail through eight indicators (involving the NP, endplate, and annulus fibrosus), with each score ranging from 0 to 2. A score of 0 indicates normal, 1–8 indicates mild degeneration, and 9–16 indicates severe degeneration.

### 2.19 Immunohistochemistry (IHC)

Following paraffin sectioning, samples were deparaffinized with xylene and graded alcohols. Antigen retrieval was performed by incubating slides with EDTA‐free trypsin (Beyotime) at 37°C for 30 min. Sections were then blocked with 5% goat serum (G1208, Servicebio) at room temperature for 30 min. After PBS washes, primary antibodies were applied and incubated overnight at 4°C, including: anti‐MMP3 (1:200, 17873‐1‐AP, Proteintech), anti‐Collagen II (1:2000, 28459‐1‐AP, Proteintech), and anti‐CD248 (1:10,000, 18160‐1‐AP, Proteintech). After three PBS washes, secondary antibodies—Goat‐anti‐mouse (G1301, Servicebio) and Goat‐anti‐rabbit (G1302, Servicebio)—were applied for 30 min. Signal visualization was achieved using DAB solution (ZLI‐9017, ZSGB‐BIO, China), followed by counterstaining with hematoxylin.

### 2.20 Statistical Analysis

Statistical analyses were performed using GraphPad Prism 9 (GraphPad Software, USA). Data from three independent experiments are presented as mean ± standard deviation (SD). Comparisons between two independent groups were analyzed using Student's *t*‐test, while one‐way ANOVA was used for multi‐group comparisons. A *p*‐value < 0.05 was considered statistically significant (*p < 0.05, **p < 0.01, ***p < 0.001, ****p < 0.0001).

## 3 Results

### 3.1 Grading of Disc Degeneration, Lactate Metabolism, and Histone Lactylation Signatures in Degenerated NP Cells

Based on the Pfirrmann grading system (Grades I–V) for IVDD, patients were classified into mild degeneration (Grade ≤ III) and severe degeneration (Grades IV–V) groups using MRI assessment (Figure 1A). Untargeted metabolomics analysis revealed active lactate metabolism in degenerated NP cells (Figure 1B), leading us to hypothesize that lactate, as the end product of glycolysis, accumulates excessively in NP tissues. Comparative analysis demonstrated significantly higher lactate levels in severely degenerated NP tissues compared to mild tissues (Figure 1C), prompting further investigation into potential lactylation modifications in the NP.

**FIGURE 1:**
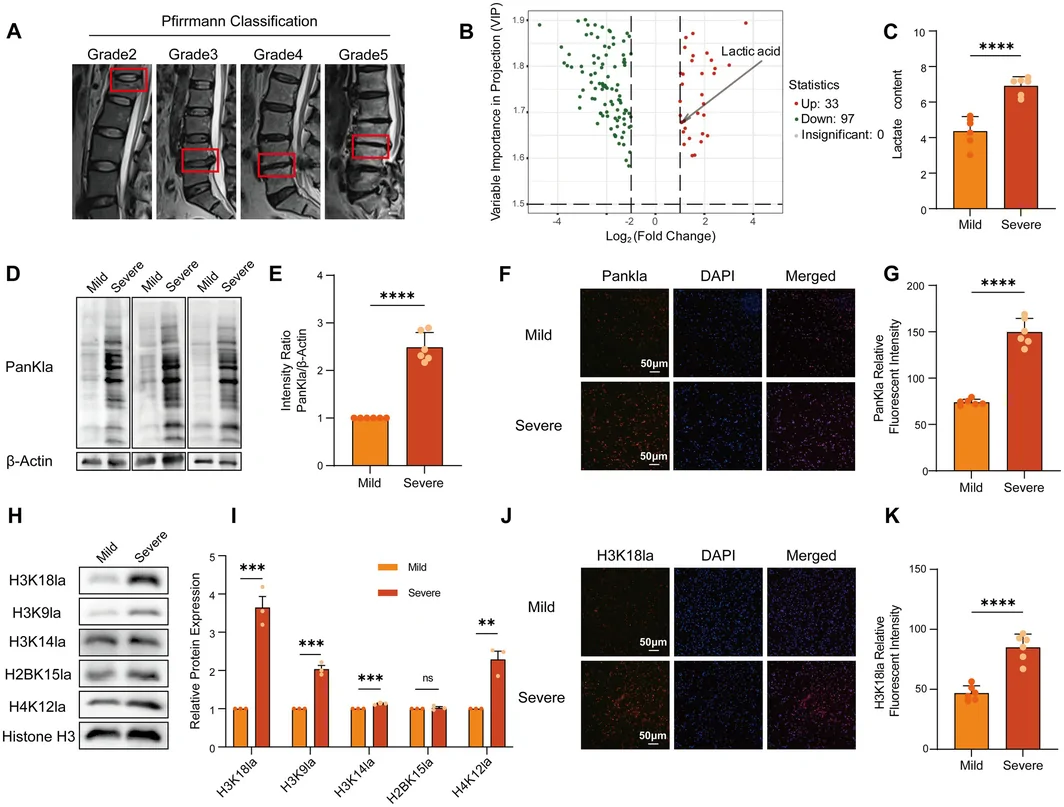
Histone lactylation in degenerated human NP cells. (A) Representative MRI images of human lumbar intervertebral discs at different degeneration stages. Discs were classified into Pfirrmann Grades 1–5. In this study, Grade 1 (healthy) NP tissues were not collected. Discs with Grade ≤ 3 degeneration were classified as mild degeneration, while Grade ≥ 4 represented severe degeneration. Red boxes indicate the surgical/lesion sites. (B) Untargeted metabolomics analysis of metabolic profiles in primary NP cells isolated from mild and severe degeneration. Differential expression analysis (volcano plot) identified lactate metabolism as prominently elevated in the severe degeneration cohort. (C) The lactate levels in the NP tissues of the mild and severe degeneration groups. Bars represent mean ± SD of six independent experiments. (D, E) Western blot analysis and quantification of global lactylation levels in NP cells from mild and severe degeneration groups. The bar graph shows normalized lactylation signals relative to β‐actin loading control. Bars represent means ± SD of six experiments. (F, G) Immunofluorescence quantification of lactylation levels in mild and severe degeneration groups. Scale bar: 50 μm. Fluorescence intensity was measured using ImageJ software. Bars represent means ± SD of six experiments. (H, I) Western blot screening and validation of differential histone lactylation sites between groups. Five established lactylation marks (H3K18la, H3K9la, H3K14la, H4K12la, and H2BK15la) were examined, with H3K18la showing the most significant changes. Bars represent means ± SD of three experiments. (J, K) Immunofluorescence quantification of H3K18la expression in NP cells. Scale bar: 50 μm. Bars represent means ± SD of six experiments. *p < 0.05, **p < 0.01, ***p < 0.001, ****p < 0.0001.

WB and IF analyses consistently showed increased global lactylation levels in degenerated human primary NP cells (Figure 1D–G). To identify specific lactylation sites, we screened five well‐characterized antibodies targeting distinct histone marks (H3K18la, H3K9la, H3K14la, H4K12la, and H2BK15la). WB results indicated that H3K18la had the most obvious changes (Figure 1H,I). This made it the focus of our subsequent studies on lactylation‐mediated regulation of NP cells. IF staining further validated elevated H3K18la levels in severely degenerated NP cells (Figure 1J,K), reinforcing its potential role in IVDD progression.

### 3.2 Lactate Increases Histone Lactylation in NP Cells, Promoting Apoptosis and ECM Degradation, While Glycolysis Inhibitors Reverse These Effects

To investigate the functional role of histone lactylation in NP cells, we first treated cells with exogenous lactate at increasing concentrations. This treatment led to a dose‐dependent increase in both global lactylation levels and specific H3K18la modifications (Figure 2A). Based on previous reports that 2‐deoxy‐d‐glucose (2DG) serves as glycolysis inhibitors, we employed this compound to suppress lactylation (Wei et al. 2024). Western blot showed that treatment with different concentrations of 2DG significantly reduced overall lactylation and H3K18la levels (Figure 2B). Scratch wound healing assays showed that lactate treatment impaired NP cell migration, and this effect was largely reversed by co‐treatment with 2DG (Figure 2C). Similarly, lactate‐mediated suppression of NP cell proliferation was effectively reversed by 2DG administration (Figure 2D). Quantitative CCK‐8 assays further demonstrated that while escalating lactate concentrations progressively inhibited NP cell growth rates, 2DG treatment promoted cellular growth (Figure 2E,F). Subsequent investigations showed that lactate exposure increased NP cell apoptosis in a concentration‐dependent manner, while simultaneously inhibiting ECM synthesis and promoting its degradation (Figure 2G,H). Importantly, inhibition of lactylation through 2DG treatment not only reduced apoptosis but also restored ECM production (Figure 2I,J).

**FIGURE 2:**
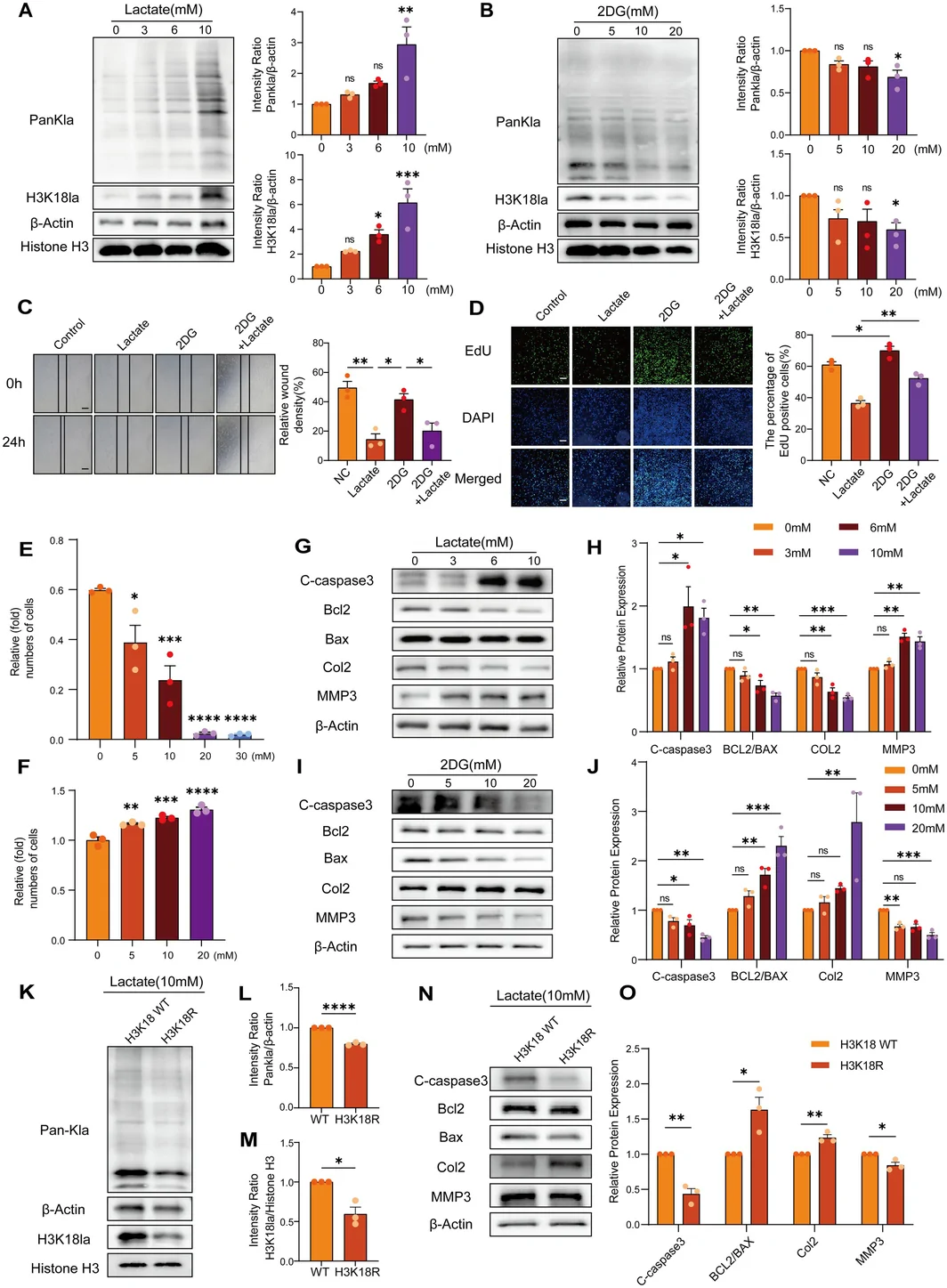
Lactate‐induced histone lactylation regulates NP cell function. (A) Western blot analysis of lactylation modifications and H3K18la expression in NP cells treated with increasing lactate concentrations (0, 3, 6, 10 mM). Quantification revealed a dose‐dependent increase in both global lactylation and H3K18la levels. (B) Dose–response effects of glycolysis inhibitors 2DG (0, 5, 10, 20 mM) on lactylation. Compounds significantly suppressed global lactylation and H3K18la in a concentration‐dependent manner. (C) Wound healing assay demonstrating 2DG (20 mM) partially rescues lactate (10 mM)‐impaired NP cell migration. Scale bar: 50 μm. (D) EdU evaluating NP cell proliferation under lactate (10 mM) and 2DG (20 mM) treatment. Scale bar: 50 μm. (E) CCK8 showing lactate dose‐dependently inhibited NP cell growth. (F) CCK8 showing 2DG dose‐dependently promoted NP cell growth. (G, H) Western blot analysis of apoptosis markers and ECM components under lactate treatment. (I, J) Western blot validation of 2DG concentration‐dependent modulation of NP cell apoptosis and ECM homeostasis. (K–M) Significant reduction in global lactylation and H3K18la levels in H3K18R mutants compared to wild‐type (WT) controls. (N, O) H3K18R mutation attenuated NP cell apoptosis while promoting ECM synthesis and inhibiting degradation. Data shown in Figure 2 are from one representative experiment (out of three independent experiments) and are presented as the mean ± SD of three biological replicates. *p < 0.05, **p < 0.01, ***p < 0.001, ****p < 0.0001.

To establish the specific contribution of H3K18la to these observed effects, we generated an H3K18R point mutant. Western blot characterization confirmed that H3K18R cells exhibited significantly reduced global lactylation and H3K18la levels compared to wild‐type controls (Figure 2K–M). Most crucially, the H3K18R mutation abrogated lactate‐induced NP cell apoptosis and ECM degradation (Figure 2N,O), providing direct evidence that these pathological changes are mediated specifically through H3K18la modifications. These comprehensive findings establish histone lactylation, particularly at H3K18, as a critical regulator of NP cell homeostasis and matrix maintenance during disc degeneration.

### 3.3 H3K18 Lactylation Regulates CD248 Expression in NP Cells

To find downstream targets of H3K18la in NP degeneration, we performed mRNA sequencing of NP cells with or without external lactate treatment (Figure 3A) and compared H3K18 WT versus H3K18R mutants (Figure 3B). Compared with the control group, external lactate upregulated 718 genes and downregulated 559 genes. Compared with WT, H3K18R upregulated 1168 genes and downregulated 379 genes. Intersection analysis identified 56 overlapping genes (Figure 3C), with CD248 as the only gene showing consistent regulation (log_2_FC > 1.5 in both datasets). CD248 was downregulated by lactate but upregulated in H3K18R, suggesting lactate suppresses CD248 expression while H3K18R rescues it.

**FIGURE 3:**
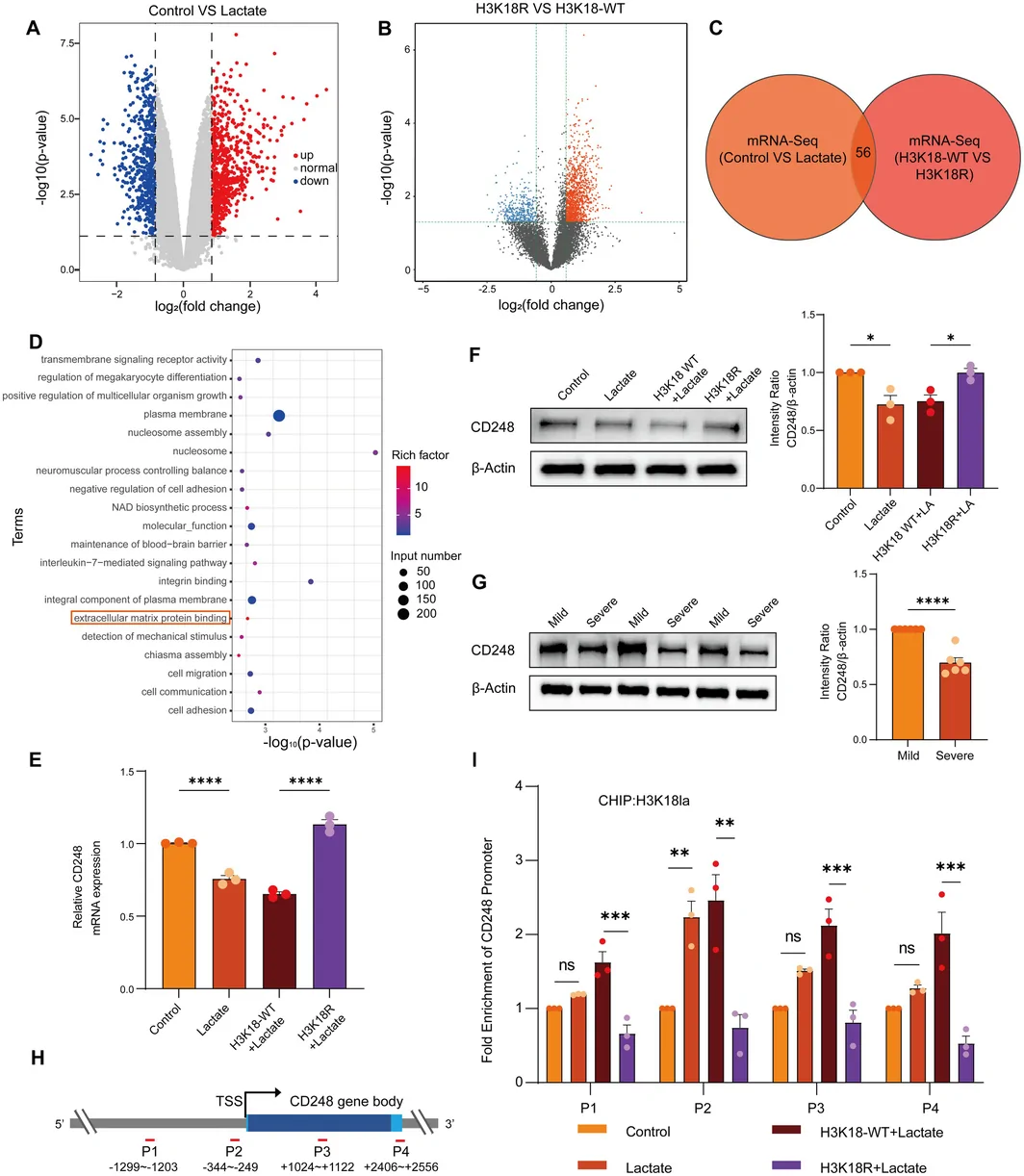
Identification and validation of CD248 as a key target regulated by H3K18la. (A) Volcano plot of RNA‐Seq analysis showing differentially expressed genes in lactate‐treated NP cells versus controls. (B) Volcano plot of RNA‐Seq analysis comparing differentially expressed genes in H3K18R versus wild‐type (WT) NP cells. (C) Intersection of differentially expressed genes identified in both sequencing datasets. (D) GO enrichment analysis revealing CD248's association with ECM protein binding. (E, F) qPCR and Western blot (WB) results confirming CD248 expression trends consistent with RNA‐Seq data across experimental groups. Bars represent means ± SD of three experiments. (G) Validation in human primary NP cells: CD248 expression was significantly reduced in the severe degeneration group (Grades IV–V). Bars represent means ± SD of six experiments. (H) Primer design (P1–P4) targeting regions near the transcriptional start site (TSS) of human CD248. (I) ChIP‐qPCR demonstrating increased H3K18la enrichment at the CD248 promoter (P2 region) following lactate treatment, while H3K18R abolished this enrichment. Bars represent means ± SD of three experiments. *p < 0.05, **p < 0.01, ***p < 0.001, ****p < 0.0001.

CD248 is involved in ECM synthesis and scar formation (Hong et al. 2024; L. Zhang et al. 2024). GO analysis linked it to “ECM protein binding” (Figure 3D), which is related to NP degeneration. Consistent with sequencing data, CD248 mRNA and protein levels decreased with lactate treatment but increased in H3K18R (Figure 3E,F). Sereve degenerated human primary NP cells also showed reduced CD248 expression (Figure 3G).

To validate H3K18la‐mediated CD248 regulation, we designed four primers near the CD248 promoter (Figure 3H). ChIP‐qPCR revealed lactate‐enhanced the enrichment of H3K18la at the CD248 promoter. Compared with WT, H3K18R showed reduced enrichment (Figure 3I). Paradoxically, histone lactylation typically activates transcription by opening chromatin or recruiting activators (D. Zhang et al. 2019). This contradicts our observation of CD248 suppression. Therefore, we hypothesized that H3K18la may recruit transcriptional repressors to inhibit CD248.

### 3.4 Lactate‐Induced H3K18la Recruits SMARCA5 to Repress CD248 Transcription and Promotes CD248 Protein Degradation via the Proteasomal Pathway

Given the paradoxical increase in H3K18la enrichment at the CD248 promoter post‐lactate treatment (contrary to observed CD248 suppression), we performed Co‐IP/MS to identify potential transcriptional repressors recruited by H3K18la. Co‐IP/MS showed that compared with the IgG control group, there were 67 significantly enriched proteins in the H3K18la‐IP group (Figure 4A). Among these, SMARCA5 (a chromatin remodeler that hydrolyzes ATP to regulate chromatin accessibility (Bomber et al. 2023)) and p53 (a transcriptional repressor that blocks Pol II binding; Y. Liu et al. 2024) were the only two transcription‐inhibitory candidates.

**FIGURE 4:**
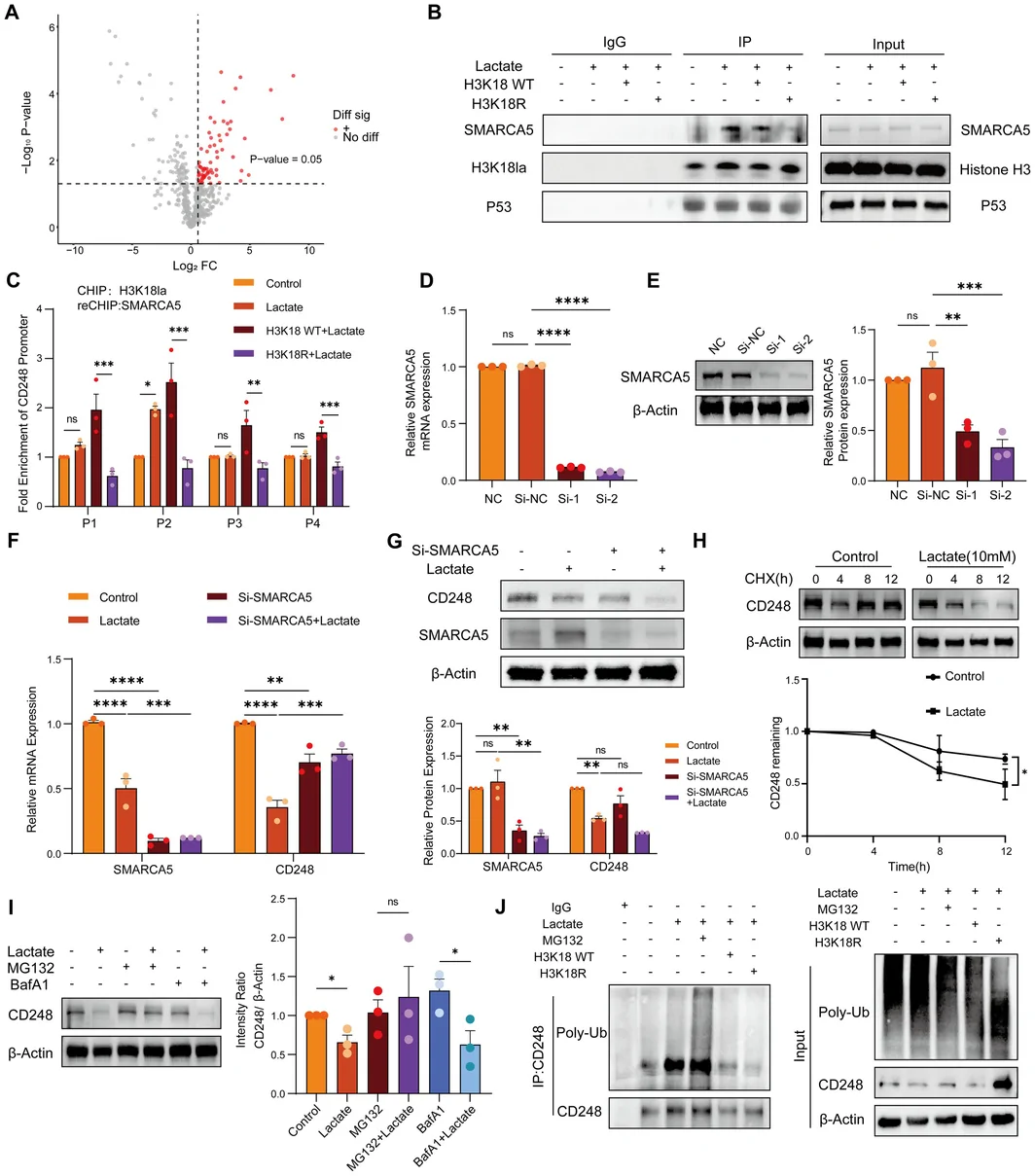
SMARCA5 mediates transcriptional repression of CD248 and lactate induces its ubiquitin–proteasome degradation. (A) Co‐immunoprecipitation mass spectrometry (Co‐IP/MS) identified 67 significantly differentially enriched proteins in the experimental group compared to controls. The data were obtained from three sets of independent biological samples. (B) ChIP‐Western blot analysis of SMARCA5 and p53 co‐precipitated with H3K18la antibody. (C) ChIP‐reCHIP‐qPCR confirmed co‐enrichment of H3K18la and SMARCA5 at the CD248 promoter upon lactate stimulation. (D, E) qPCR and Western blot validation of SMARCA5 knockdown efficiency (si‐SMARCA5). (F) qPCR demonstrated that si‐SMARCA5 significantly restored CD248 transcriptional levels suppressed by lactate. (G) At the protein level, si‐SMARCA5 failed to reverse lactate‐induced CD248 suppression. (H) Cycloheximide (CHX) chase assay showed accelerated CD248 degradation in lactate‐treated cells versus controls across time points. (I) Proteasome inhibitor MG132 effectively blocked CD248 degradation, while autophagy‐lysosome inhibitor BafA1 showed no effect. (J) Co‐IP analysis quantified CD248 ubiquitination levels across experimental groups. Data in Figure 4 are shown as means ± SD of three biological replicates. *p < 0.05, **p < 0.01, ***p < 0.001, ****p < 0.0001.

ChIP‐WB confirmed SMARCA5 and p53 presence in H3K18la immunoprecipitates (Figure 4B). While p53 levels showed no group‐specific differences, SMARCA5 was significantly elevated in the lactate‐treated and H3K18‐WT groups. This indicates that H3K18la recruits SMARCA5 in a lactate‐dependent manner. ChIP‐reChIP‐qPCR further demonstrated co‐enrichment of H3K18la and SMARCA5 near the CD248 promoter upon lactate stimulation (Figure 4C).

SMARCA5 knockdown (si‐SMARCA5) in NP cells (Figure 4D,E) partially rescued lactate‐induced CD248 transcriptional repression (Figure 4F) but failed to restore CD248 protein levels (Figure 4G), suggesting additional post‐translational regulation. Cycloheximide (CHX) chase assays revealed a shorter CD248 half‐life in lactate‐treated cells (Figure 4H), while the proteasome inhibitor MG132 reversed CD248 depletion (Figure 4I), confirming lactate promotes CD248 degradation via the ubiquitin–proteasome pathway (UPP), not autophagy.

Co‐IP validated that lactate enhances CD248 ubiquitination, which accumulates with MG132 treatment and diminishes in H3K18R mutants (Figure 4J). Thus, H3K18la mediates CD248 protein degradation through UPS activation.

### 3.5 Lactate‐Suppressed CD248 Promotes NP Cells Apoptosis and ECM Degradation In Vitro

Since CD248 is regulated by H3K18la and shows reduced expression in degenerated NP cells, we next investigated the functional role of CD248 in NP cells. We transfected NP cells with si‐CD248 to knock down CD248 expression. Compared to Si‐NC groups, both mRNA and protein levels of CD248 were significantly reduced in si‐CD248‐treated NP cells (Figure 5A–C). Furthermore, si‐CD248 markedly promoted NP cell apoptosis, enhanced ECM degradation, and inhibited ECM synthesis (Figure 5A,B). Scratch assays demonstrated that NP cell migration capacity was significantly weakened in the si‐CD248 group compared to Si‐NC after 24 h (Figure 5D,E). EdU and CCK‐8 assays revealed that si‐CD248 significantly suppressed NP cell proliferation (Figure 5F–H).

**FIGURE 5:**
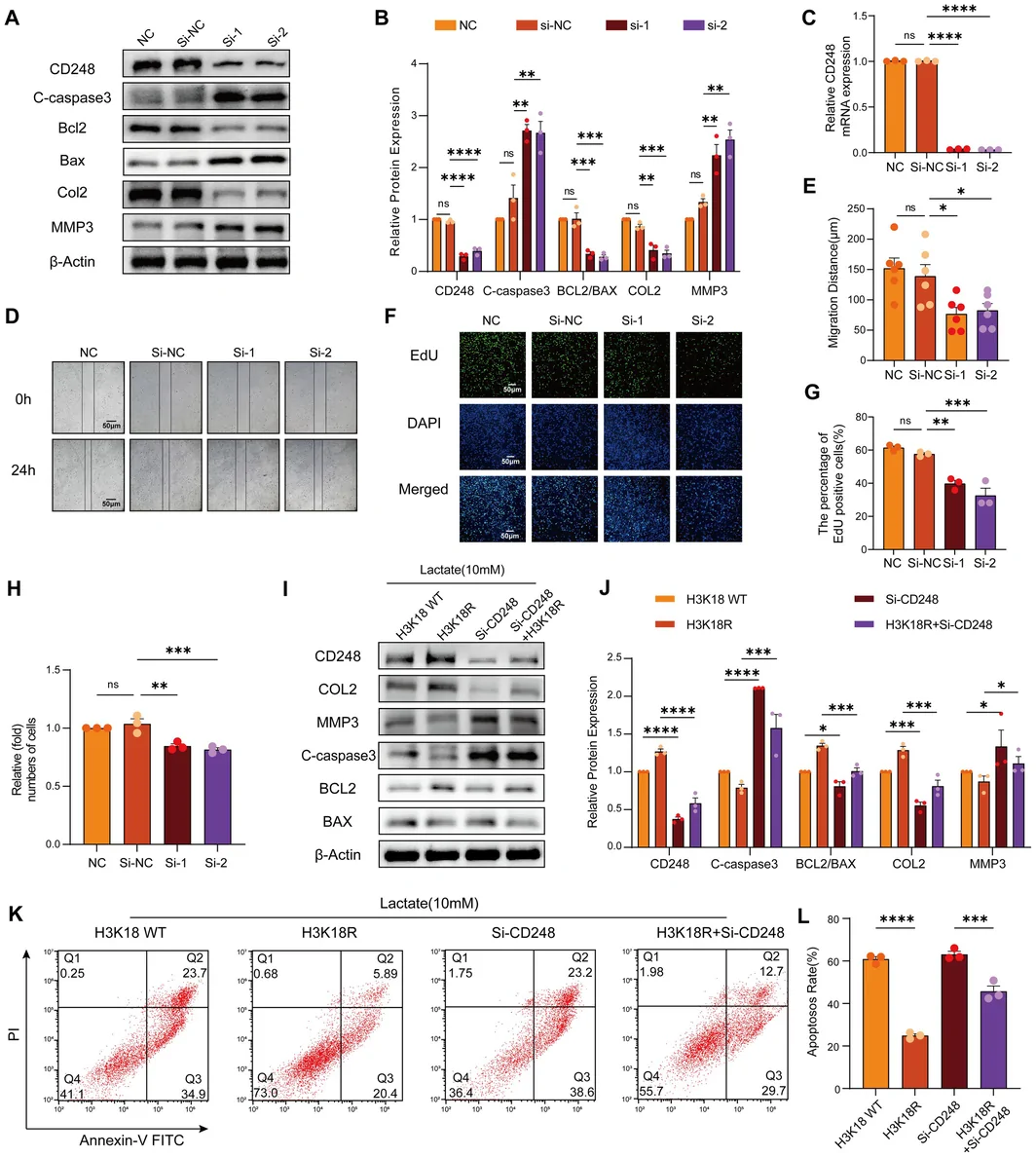
Functional impacts of CD248 knockdown on NP cell. (A, B) Western blot analysis and quantification of apoptosis‐related and ECM markers in NP cells following si‐CD248 treatment. (C) qPCR validation of CD248 mRNA knockdown efficiency by si‐CD248. (D, E) Scratch assay evaluating NP cell migration capacity post si‐CD248 transfection. *n* = 6. (F, G) EdU assay assessing NP cell proliferation after si‐CD248 intervention. (H) CCK‐8 analysis of si‐CD248 effects on NP cell viability. (I, J) H3K18R transfection rescued si‐CD248‐induced NP cell apoptosis and ECM degradation. (K, L) Flow cytometric analysis of NP cell apoptosis rates. Data in Figure 5 are shown as means ± SD of three biological replicates. *p < 0.05, **p < 0.01, ***p < 0.001, ****p < 0.0001.

We then examined whether introducing the H3K18R mutation after si‐CD248 treatment could reverse the pro‐apoptotic effects of si‐CD248 and its negative regulation of ECM. Western blot analysis revealed that after si‐CD248 treatment, the addition of H3K18R reversed the transcriptional inhibition on CD248, and the protein expression level of CD248 increased. Compared with the si‐CD248 group, this had a partial inhibitory effect on the apoptosis of NP cells and the degradation of ECM (Figure 5I,J). Flow cytometry results indicated that the H3K18 WT  and si‐CD248 group exhibited higher apoptosis levels, while H3K18R significantly ameliorated NP cell apoptosis (Figure 5K,L).

### 3.6 CD248 Overexpression Regulates NP Cell Apoptosis and ECM Synthesis Through the Hedgehog Pathway

Following successful CD248 overexpression (CD248‐OE) (Figure 6A), compared to the control group, CD248‐OE demonstrated inhibitory effects on NP cell apoptosis, promoted ECM synthesis, and suppressed ECM degradation. In scratch assays (Figure 6B), CD248‐OE enhanced NP cell migration capacity. We further examined whether CD248‐OE could reverse lactate‐induced NP cell apoptosis and ECM synthesis inhibition. The results showed that after CD248‐OE, lactate‐induced NP cell apoptosis was reversed, and lactate‐mediated ECM degradation was also counteracted (Figure 6C,F).

**FIGURE 6:**
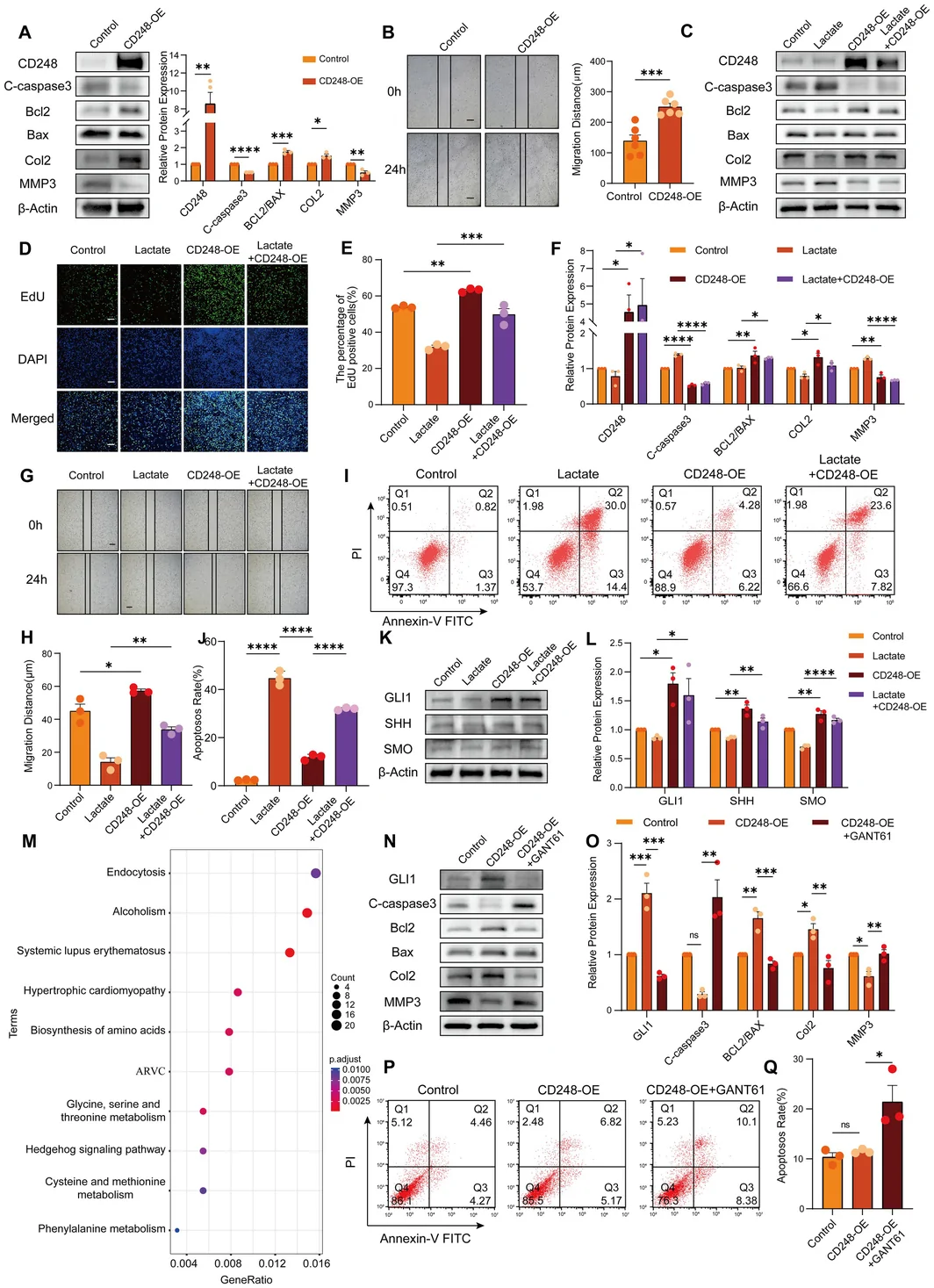
CD248 attenuates lactate‐induced NP cell degeneration via the Hedgehog pathway. (A) Validation of OE‐CD248 effects on NP cell apoptosis and ECM synthesis. (B) Scratch assay at 24 h demonstrated enhanced NP cell migration capacity following CD248‐OE. (C, F) Under elevated lactate conditions, CD248‐OE ameliorated lactate‐induced NP cell apoptosis and ECM degradation. (D, E) EdU assay confirmed improved NP cell proliferation with CD248‐OE under lactate treatment. (G, H) CD248‐OE restored NP cell migration capacity under high lactate conditions. (I, J) CD248‐OE suppressed NP cell apoptosis. (M) KEGG analysis of transcriptome sequencing data highlighted the Hedgehog pathway's significant involvement. (K, L) CD248‐OE activated the Hedgehog pathway, while lactate inhibited this activation. (N, O) Treatment with the Hedgehog pathway inhibitor GANT61 confirmed CD248's functional dependence on this pathway. (P, Q) Inhibition of the Hedgehog pathway by GANT61 promoted apoptosis in NP cells. Data in Figure 6 are shown as means ± SD of three biological replicates. *p < 0.05, **p < 0.01, ***p < 0.001, ****p < 0.0001.

EdU assays revealed (Figure 6D,E) that CD248‐OE improved NP cell proliferation under lactate treatment. Scratch assay results demonstrated (Figure 6G,H) that lactate inhibits the migration of NP cells, while CD248‐OE can alleviate this situation. Flow cytometry analysis confirmed that CD248‐OE significantly reversed lactate‐elevated NP cell apoptosis (Figure 6I,J).

In previous transcriptome sequencing (Figure 6M), KEGG pathway enrichment analysis indicated significant enrichment of the Hedgehog pathway. Studies have reported that the Sonic Hedgehog pathway can effectively inhibit NP cell apoptosis to prevent IVDD (Bao et al. 2021; L. Liu et al. 2023). We therefore hypothesized that lactate suppresses CD248 expression, thereby inhibiting the Hedgehog pathway to promote NP cell apoptosis and ECM degradation. Western blot results (Figure 6K,L) showed that lactate downregulated Hedgehog pathway core proteins (GLI1, SHH, and SMO), while CD248‐OE restored their expression, confirming our hypothesis.

Subsequently, we treated CD248‐overexpressing NP cells with GANT61, a specific GLI1 inhibitor. Western blot (Figure 6N,O) demonstrated that GANT61 effectively reduced CD248‐OE‐elevated GLI1 levels and increased CD248‐OE‐suppressed CAS3 expression. Similarly, flow cytometry results showed that GANT61 treatment significantly promoted NP cell apoptosis (Figure 6P,Q). These findings collectively indicate that CD248 inhibits NP cell apoptosis and promotes ECM synthesis by activating the Hedgehog pathway.

### 3.7 In Vivo Experiments

To validate the effects of lactylation modification and CD248 on NP cell degeneration, we established a rat tail IVDD model using needle puncture and evaluated the outcomes in vivo. After model establishment, the experimental group received intradiscal injections of AAV9‐CD248‐OE (titer: 1.70 × 10^13^ vg/mL). Rats were sacrificed 6 weeks after modeling.

Masson's trichrome, safranin O‐fast green, and H&E staining revealed significant structural disruption in punctured discs (Figure 7A,B). Although the treatment group had NP tissue damage, the NP, annulus fibrosus, and cartilage endplates maintained a relatively normal structure (Figure 7A,B). Western blot analysis of rat NP tissue proteins yielded consistent results (Figure 7C,D), indicating that CD248‐OE markedly alleviates IVDD progression. Immunohistochemistry showed reduced COL2 and increased MMP3 in degenerated NP tissues, whereas the CD248‐OE group demonstrated significantly higher COL2 expression compared to the degeneration group (Figure 7E,F).

**FIGURE 7:**
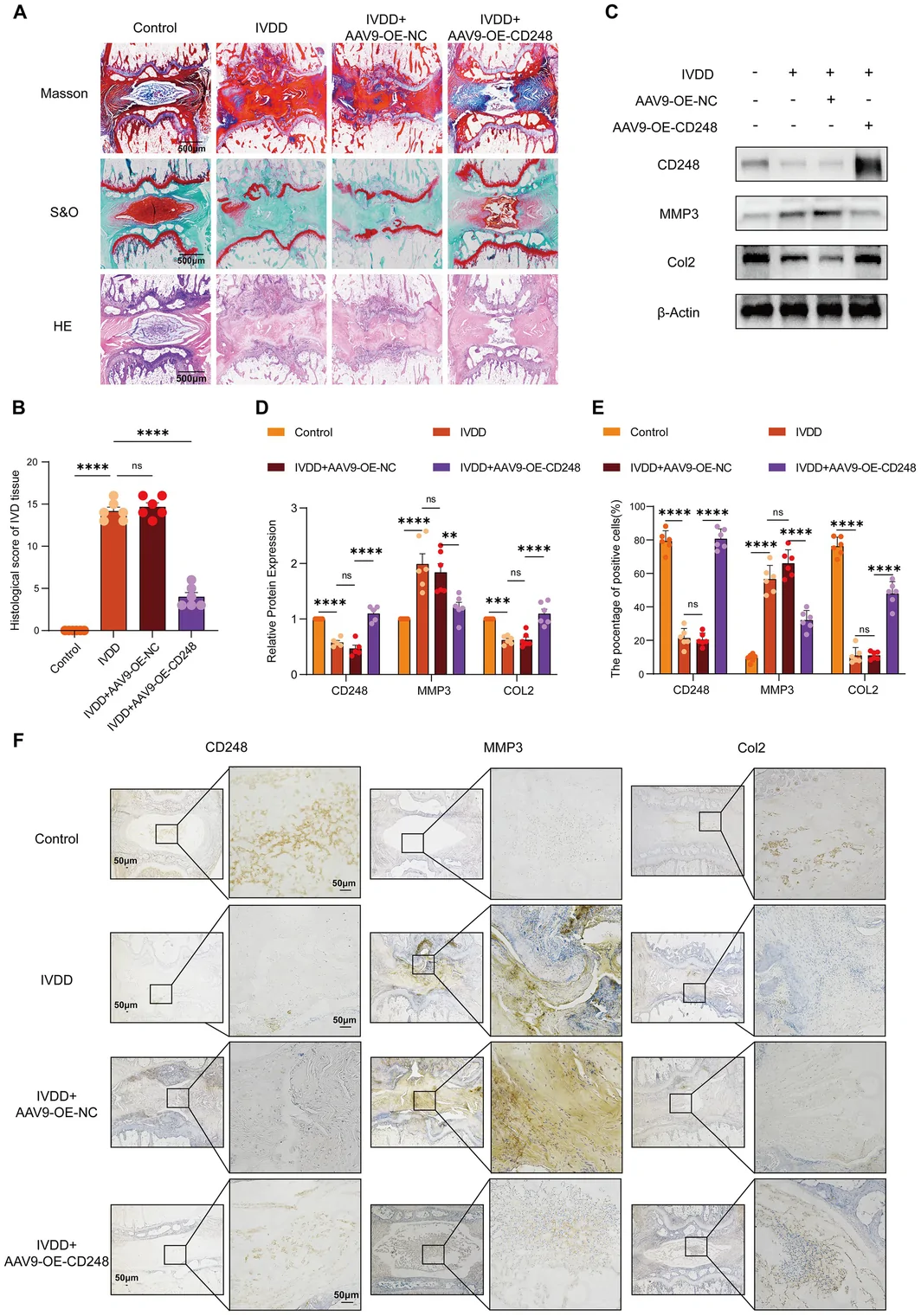
In vivo validation of the therapeutic efficacy in a rat IVDD model. (A) Masson's trichrome, safranin O (S&O), and H&E staining of intervertebral discs from Control, IVDD, IVDD+AAV9‐OE‐NC, and IVDD+AAV9‐OE‐CD248 groups. Scale bar: 500 μm. (B) Histological staining score of rat intervertebral disc tissue. (C, D) Western blot analysis of protein extracts from rat tail intervertebral disc tissues in all four groups. (E) Immunohistochemical staining score of rat intervertebral disc tissue. (F) IHC staining of intervertebral disc tissue sections from the four groups. Scale bar: 50 μm. The in vivo data in Figure 7 were obtained from six independent biological replicates. *p < 0.05, **p < 0.01, ***p < 0.001, ****p < 0.0001.

## 4 Discussion

In spinal disorders, IVDD ranks among the most prevalent conditions. Degenerated discs exhibit an aberrant microenvironment, characterized by elevated extracellular osmotic pressure, acidic conditions, and nutrient deficiency (Adams and Roughley 2006; Leung et al. 2006; Sakai 2008). Zhao et al. (2021) demonstrated that exposure of human NP cells to 6 mM lactate activates the NLRP3 inflammasome and promotes ECM degeneration. As human NP tissue lacks vascular supply, anaerobic glycolysis becomes the primary energy source, leading to lactate accumulation.

In this study, we confirmed elevated lactate metabolism and histone lactylation (specifically at H3K18) in degenerated NP cells. Exogenous lactate supplementation mimicking acidic conditions increased lactylation, while glycolysis inhibitor (2‐DG) suppressed it by blocking lactate production. Prior studies reported that enhanced lactylation promotes apoptosis (Weng et al. 2025; Xu et al. 2024) and impairs ECM synthesis (Lan et al. 2025), which our data corroborated. Through H3K18 point mutation (K → R), we observed reduced global lactylation, with H3K18R significantly inhibiting NP cell apoptosis and ECM degradation. These findings show that lactate makes disc degeneration worse through H3K18la. Inhibiting this lactylation modification can slow down the degeneration process, and this provides new ways to prevent IVDD.

CD248, also known as Endosialin or Tumor Endothelial Marker 1 (TEM1), is a single‐pass transmembrane glycoprotein containing a C‐type lectin‐like domain (Khan et al. 2019). Initially identified in vascular endothelial cells of certain cancers (Rettig et al. 1992), subsequent studies revealed its expression in stromal fibroblasts and pericytes within tumor microenvironments, where it contributes not only to tumor progression but also to tissue repair processes (Bagley et al. 2008; Christian et al. 2008; Di Benedetto et al. 2019). CD248 plays a role in tissue remodeling and is associated with enhanced stromal cell proliferation and migration (Teicher 2019). Research indicates that CD248 is highly expressed in osteosarcoma, where its interaction with fibronectin (FN) promotes tumor cell migration (Kondo et al. 2022).

In our study, GO analysis demonstrated that CD248's protein functions are primarily associated with developmental processes, cell proliferation, and ECM regulation—key pathways implicated in NP degeneration. We confirmed that during NP cell degeneration, elevated lactate levels enable H3K18la to modulate CD248 transcription and protein expression. However, ChIP‐qPCR yielded unexpected results: while lactate increased H3K18la enrichment at the CD248 promoter, both transcriptome sequencing and qPCR data showed lactate‐mediated suppression of CD248 mRNA expression, contradicting prior reports (Wei et al. 2024; D. Zhang et al. 2019). This discrepancy led us to hypothesize that H3K18la might recruit transcriptional repressors to inhibit CD248 expression.

Co‐IP/MS results revealed an association between H3K18la, SMARCA5, and p53. SMARCA5, a chromatin remodeling complex, is a critical enzyme with nucleosome repositioning activity (Kokavec et al. 2017). Dynamic changes in chromatin configuration play a pivotal role in gene expression regulation. Chromatin remodelers hydrolyze ATP to slide nucleosomes, altering chromatin structure and accessibility, while cooperating with histones to facilitate histone variant exchange. Thus, these enzymatic complexes possess the capacity to regulate all DNA‐templated cellular processes (Bomber et al. 2023).

As a classical transcriptional repressor, p53 primarily functions by directly binding gene promoters or enhancers or by recruiting other repressors to suppress transcription (Y. Liu et al. 2024). ChIP‐Western blot results demonstrated that, unlike SMARCA5, p53 expression remained unaffected by experimental interventions, showing no significant differences across the four groups. In contrast, SMARCA5 enrichment markedly increased in both the exogenous lactate‐treated group and H3K18 WT cells. Subsequent ChIP‐reChIP‐qPCR analysis confirmed that lactate‐induced H3K18la recruits SMARCA5 to the CD248 promoter region. This finding explains the paradoxical observation wherein lactate suppresses CD248 transcription despite increased H3K18la enrichment at its promoter.

Following SMARCA5 knockdown (si‐SMARCA5), CD248 mRNA levels were restored, but protein expression remained unchanged, suggesting SMARCA5 primarily regulates CD248 transcription rather than protein stability. We thus investigated whether lactate affects CD248 protein degradation through alternative pathways. CHX chase assays confirmed that lactate accelerates CD248 protein degradation compared to controls.

Given that protein degradation in degenerative diseases primarily occurs via the UPP or autophagy‐lysosome pathway (Clague and Urbé 2010), we treated cells with the proteasome inhibitor MG132 or the autophagy‐lysosome inhibitor BafA1. MG132 treatment reversed lactate‐induced CD248 degradation and promoted ubiquitinated protein accumulation, while H3K18R significantly reduced ubiquitination levels.

The UPP serves as the primary non‐lysosomal proteolytic pathway, regulating critical cellular functions—including cell cycle progression, DNA repair, growth, and differentiation (Fukasawa et al. 2012). Dysregulation of this system disrupts cellular homeostasis and contributes to diseases such as cancer, degenerative disorders, and fibrotic conditions. Our results demonstrate that H3K18la promotes CD248 protein degradation via the UPP under high lactate conditions. This reveals a dual regulatory mechanism in NP cell degeneration: lactate controls CD248 expression through transcriptional (SMARCA5‐mediated) and post‐translational (UPP‐dependent) modifications.

The Hedgehog (Hh) signaling pathway plays a critical role in vertebrate embryonic development and tumorigenesis (Bangs and Anderson 2017). In mammals, Hh signaling is essential for organogenesis, tissue regeneration, and homeostasis in nearly all organs. Its core components include Hedgehog ligands, receptor proteins, and downstream effector molecules (Fattahi et al. 2018).

NP cells originate from the embryonic notochord. Studies have shown that Sonic hedgehog (Shh) signaling from NP cells controls their growth and differentiation during murine development (Dahia et al. 2012). Our research revealed that lactate inhibits Hh pathway activation, while CD248 overexpression (CD248‐OE) counteracts this inhibition and promotes downstream effector GLI1 activation. By using GANT61, a specific GLI1 inhibitor, we confirmed that CD248 suppresses NP cell apoptosis and enhances ECM synthesis through Hh pathway activation, thereby ameliorating NP cell degeneration.

In summary, our study demonstrates that elevated lactate levels in degenerated intervertebral discs induce histone lactylation modifications in NP cells, with H3K18 identified as the specific modification site. Exogenous lactate supplementation further confirmed that increasing lactate concentrations enhance NP cell lactylation, while glycolysis inhibitor 2‐DG effectively suppresses this modification. We further revealed that H3K18la regulates CD248 expression through a dual mechanism: (1) at the transcriptional level by recruiting the transcriptional repressor SMARCA5 to inhibit CD248 mRNA synthesis, and (2) at the protein level by promoting CD248 degradation via the UPP. Combined with transcriptome sequencing data and existing literature, we validated that CD248 modulates NP cell apoptosis and ECM synthesis through activation of the Hedgehog pathway.

Due to the relatively hypoxic environment of the intervertebral disc, it can obtain energy almost exclusively through anaerobic glycolysis. Therefore, inhibiting glycolysis in the complex overall environment of the intervertebral disc may not yield satisfactory therapeutic effects (such as long‐term use of 2‐DG). This might damage the basic energy metabolism and survival ability of the cells. In this experiment, the short‐term effect of 2‐DG is more of a tool for mechanism research rather than a direct treatment plan. In the future, while ensuring the basic metabolism of the NP cells, intervening in CD248 can more precisely play a role in delaying IVDD.

Our study has some limitations. First, due to cost constraints, we did not perform comprehensive LC–MS/MS analysis for lactylation site screening. Instead, we selected five well‐studied histone sites with commercially available antibodies based on literature review. Second, the mechanistic details of how lactate promotes CD248 degradation through the proteasomal pathway require further investigation to fully elucidate the underlying molecular events. Finally, juvenile rats aged 6–8 weeks are still in the periadolescent stage and retain a notochordal cell‐rich NP; therefore, this puncture model cannot fully recapitulate the cellular composition or chronic natural history of adult human IVDD.

## Author Contributions

Y.M. conceived and designed the study. Z.Y. supervised the research. Y.M., T.S., and Q.G. performed the experiments. Y.M. analyzed the data and wrote the manuscript. W.Z., S.H., S.L., and T.S. contributed to the animal experiments. J.G. and Z.Y. edited the paper. All authors read and approved the final manuscript.

## Funding

This research was supported by the Horizontal Project of Harbin Medical University (22992230114).

## Ethics Statement

This study was conducted in accordance with the Declaration of Helsinki and approved by the Ethics Committee of First Affiliated Hospital of Harbin Medical University (No. 202573). All experimental protocols were performed in compliance with relevant guidelines and regulations.

## Consent

Written informed consent was obtained from all participants or their legal guardians prior to study enrollment.

## Conflicts of Interest

The authors declare no conflicts of interest.

## Supporting information


**Data S1:** Supplementary antibodies.

## Data Availability

The RNA sequencing data that support the findings of this study are openly available in NCBI Sequence Read Archive (SRA) under the Bioproject accession, reference number 1434230 and 1434036. The mass spectrometry proteomics data have been deposited to the ProteomeXchange Consortium (https://proteomecentral.proteomexchange.org) via the iProX partner repository with the dataset identifier PXD076429.
