# Supplementary material for: H3K18 Lactylation in Degenerated Nucleus Pulposus Cells Suppresses CD248 Through Transcriptional and Post‐Translational Modifications to Promote Apoptosis and Extracellular Matrix Degradation
Source: Aging Cell. 2026 Nov 10;25(11):e70810. doi: 10.1111/acel.70810 (PMC13751322; doi:10.1111/acel.70810)
Supplement: Supplementary file 1 [file ACEL-25-e70810-s001.docx]

| **Antibodies** | **Source** | **Identifier** | **RRID** |
| --- | --- | --- | --- |
| anti-β-Actin | Proteintech | 66009-1-Ig | AB_2687938 |
| anti-Histone H3 | Proteintech | 17168-1-AP | AB_2716755 |
| anti- H3K18la | PTMbio | PTM-1427RM | AB_3076698 |
| anti-Pankla | PTMbio | PTM-1401RM | AB_2942013 |
| anti-H3K9la | PTMbio | PTM-1419RM | AB_3076695 |
| anti-H3K14la | PTMbio | PTM-1414RM | AB_3076697 |
| anti-H4K12la | PTMbio | PTM-1411RM | AB_2941896 |
| anti-H2BK15la | PTMbio | PTM-1426RM | AB_3713165 |
| anti-Cleaved Caspase 3 | Proteintech | 25128-1-AP | AB_3073913 |
| anti-bax | Proteintech | 50599-2-Ig | AB_2061561 |
| anti-bcl2 | Proteintech | 12789-1-AP | AB_2227948 |
| anti-MMP3 | Proteintech | 17873-1-AP | AB_2146587 |
| anti-Colleagen2 | Proteintech | 28459-1-AP | AB_2881147 |
| anti-CD248 | Proteintech | 18160-1-AP | AB_2073200 |
| anti-SMARCA5 | Proteintech | 13066-1-AP | AB_2191854 |
| anti-ubiquitin | Proteintech | 10201-2-AP | AB_671515 |
| anti-P53 | HUABIO | SA39-07 | AB_3069595 |
| HRP-conjugated Goat Anti-Rabbit IgG | Proteintech | SA00001-2 | AB_2722564 |
| HRP-conjugated Goat Anti-Mouse IgG | Proteintech | SA00001-1 | AB_2722565 |
